# Current Perspectives on the Inflammatory Bowel Disease Pathogenesis of Microbiota and the Gut-Brain Axis, and Emerging Therapeutics

**DOI:** 10.3390/biomedicines14040859

**Published:** 2026-04-09

**Authors:** Yujia Lin, Panpan Lu, Qiang Ding, Mei Liu

**Affiliations:** Department of Gastroenterology, Tongji Hospital, Tongji Medical College, Huazhong University of Science and Technology, Wuhan 430030, China; linyujia0217@163.com (Y.L.); lu2020tj5085@126.com (P.L.); dingqiang@tjh.tjmu.edu.cn (Q.D.)

**Keywords:** inflammatory bowel disease, gut microbiota, pathogenesis, gut–brain axis, microbiota-targeted therapy

## Abstract

The pathogenesis of inflammatory bowel disease (IBD) is driven by an interplay among intestinal dysbiosis and aberrant mucosal immune responses. This review centers on the microbiota as a pivotal pathogenic hub, systematically dissecting how three hallmark features of dysbiosis—reduced microbial alpha diversity, depletion of immunomodulatory commensals, and expansion of pro-inflammatory pathobionts—collectively compromise epithelial barrier function, promote bacterial translocation, and sustain chronic mucosal inflammation. We further integrate emerging evidence implicating bidirectional gut-brain axis communication in amplifying both peripheral inflammation and central nervous system (CNS)-mediated behavioral comorbidities. Building on this mechanistic framework, we critically evaluate next-generation microbiota-targeted interventions: standardized fecal microbiota transplantation (FMT), rationally designed live biotherapeutic products (LBPs), precision phage cocktails targeting defined pathobionts, and microbiome-informed dietary strategies. Collectively, these approaches represent a paradigm shift—from broad-spectrum immunosuppression toward mechanism-guided, ecosystem-level modulation—thereby advancing the goal of precision medicine in IBD.

## 1. Introduction

Inflammatory bowel disease (IBD) is a group of immune-mediated disorders characterized by chronic, relapsing intestinal inflammation, primarily encompassing Crohn’s disease (CD) and ulcerative colitis (UC) [1]. Clinically, IBD presents with abdominal pain, diarrhea, and systemic symptoms such as fatigue and weight loss [2,3], with disease-specific manifestations reflecting differences in the extent and depth of intestinal involvement [3,4,5]. Beyond intestinal pathology, a substantial proportion of patients develop extraintestinal manifestations affecting the joints, skin, liver, and eyes [6].

Over recent decades, the global burden of IBD has continued to rise, expanding from traditionally high-incidence regions in Western Europe and North America to newly industrialized countries, thereby forming a so-called ‘Westernized’ epidemiological pattern [7,8]. IBD can occur at any age, with a peak incidence in early adulthood and an increasing proportion of pediatric-onset and elderly-onset cases, underscoring the challenge of lifelong disease management [9,10]. It is worth noting that sex-related differences in disease distribution have been observed, with age-dependent shifts in predominance between CD and UC. Specifically, CD shows a transition from male predominance in childhood to female predominance in adulthood, whereas UC demonstrates a relative shift toward male predominance in later life [11,12]. These trends underscore the importance of understanding the underlying mechanisms that drive disease onset and progression.

IBD is now widely recognized as a multifactorial disorder arising from complex interactions among host genetics, environmental factors, immune dysregulation, and the gut microbiota [13]. Among these, gut microbiota dysbiosis has emerged as a central pathogenic hub, linking environmental exposures to aberrant mucosal immune responses. Hallmark features of dysbiosis—including reduced microbial diversity, depletion of immunoregulatory commensals, and expansion of pro-inflammatory pathobionts—collectively disrupt epithelial barrier integrity, promote bacterial translocation, and sustain chronic intestinal inflammation [14]. These alterations not only reshape local immune responses but also influence systemic immune homeostasis. In parallel, increasing attention has been directed toward the gut–brain axis (GBA) as a key regulatory network in IBD. Bidirectional communication between the CNS and the gastrointestinal tract—mediated through neural, endocrine, and immune pathways—modulates intestinal permeability, microbial composition, and inflammatory responses [15]. Psychological stress and neuroendocrine signaling can exacerbate intestinal inflammation, while microbiota-derived metabolites can influence brain function. Together, these interactions form an integrated neuro–immune–microbial axis that contributes to both intestinal pathology and extraintestinal comorbidities.

Despite advances in diagnostic strategies, significant challenges remain in accurately predicting disease course and therapeutic response. Colonoscopy with histological assessment remains the gold standard for diagnosis [16], supported by cross-sectional imaging and non-invasive biomarkers. However, conventional approaches are increasingly complemented by emerging technologies. Notably, artificial intelligence (AI)-assisted endoscopy is rapidly transforming IBD diagnostics, enabling real-time detection of mucosal inflammation, automated disease activity scoring, and prediction of histologic remission [17,18,19]. In addition, non-invasive imaging modalities such as intestinal ultrasound are gaining prominence for disease monitoring [20]. Biomarker research in IBD has evolved from single-analyte approaches to integrated, multi-omics-based models [21]. Fecal calprotectin remains the most widely used non-invasive marker for assessing intestinal inflammation and correlates closely with endoscopic disease activity. Importantly, dynamic changes in fecal calprotectin levels—particularly reductions following induction therapy with biologics—have demonstrated prognostic value in predicting sustained remission and treatment response [18,22]. Emerging biomarkers, including proteomic, transcriptomic, and microbiome-derived signatures, combined with machine learning approaches [23], are increasingly enabling precise disease stratification and early diagnosis.

Collectively, these advances support a paradigm shift from symptom-based management toward mechanism-oriented therapeutic strategies in IBD. In this context, effective treatment increasingly requires alignment with key pathogenic processes, including microbiota alterations, and neuro–immune interactions along the GBA. Accordingly, this review focuses on the integrated mechanisms underlying IBD pathogenesis, with particular emphasis on microbiota dysbiosis as a central pathogenic axis, while also addressing the role of the GBA. We further summarize established immune-targeted therapies and emerging microbiota-based and mechanism-driven interventions, aiming to provide a conceptual framework for precision medicine in IBD.

## 2. Pathogenesis

The pathogenesis of IBD arises from dynamic, reciprocal interactions among host genetic susceptibility, dysregulated mucosal immunity, and environmental triggers—including microbial exposures [13]. Central to this process are four interlocking pathological axes: (i) aberrant activation of both innate and adaptive immune effectors—particularly Th1 and Th17 lymphocytes—driving excessive production of pro-inflammatory cytokines such as tumor necrosis factor-α (TNF-α), interferon-γ (IFN-γ), and interleukin-17 (IL-17) [24]; (ii) progressive breakdown of intestinal epithelial barrier integrity, characterized by structural disassembly of tight junctions, increased epithelial apoptosis, and impaired restitution [25]; (iii) profound dysbiosis of the gut microbiota, marked by depletion of commensal taxa, expansion of pathobionts, and reduced alpha diversity [26]; and (iv) neuroimmune dysregulation along the GBA, wherein stress, autonomic dysfunction, and enteric neural signals modulate intestinal inflammation by altering gut motility, epithelial secretion, and immune cell reactivity, thereby establishing a bidirectional conduit between the CNS and the inflamed gut microenvironment [27,28]. Critically, dysbiosis is not merely an epiphenomenon but serves as a permissive “ecological background” that compromises barrier resilience and skews immune homeostasis—thereby heightening host vulnerability to specific pathogens. In turn, infection with defined enteric pathogens acts as a potent “molecular trigger”: their virulence factors directly disrupt epithelial architecture, activate pattern-recognition receptors, and amplify inflammatory cascades [29]. This interconnected network is further shaped by brain–gut signals, which can lower the threshold for immune activation and influence the trajectory of dysbiosis, creating a self-reinforcing system. These elements engage in a self-reinforcing tripartite loop—dysbiosis facilitates pathogen colonization and immune dysregulation; pathogen invasion exacerbates dysbiosis and intensifies inflammation; and chronic inflammation further damages the barrier and reshapes the microbial niche—ultimately sustaining a state of non-resolving, tissue-destructive inflammation, with the GBA acting as a modulatory amplifier that integrates psychosocial and neuroendocrine inputs into the core pathogenic circuitry [28,30,31].

### 2.1. Pathogen Infection

Intestinal pathogenic microbial infections represent significant risk factors for IBD onset and relapse, as confirmed in multiple clinical studies [32]. Systematic characterization and discussion of the molecular mechanisms employed by these pathogens may provide further insight into IBD pathogenesis and inform novel and therapeutic strategies.

#### 2.1.1. Bacterial Pathogens

##### *Clostridioides difficile* 

The principal virulence factors of *Clostridioides difficile* are two exotoxins, toxin A (TcdA) and toxin B (TcdB), encoded by the *tcdA* and *tcdB* genes [33]. Both toxins function as glucosyltransferases that inactivate host Rho and Ras family GTPases through glucosylation. This modification leads to depolymerization of actin microfilaments, collapse of the cytoskeletal architecture, and disruption of tight junctions between intestinal epithelial cells. Consequently, epithelial barrier integrity is damaged, resulting in increased permeability and luminal fluid accumulation [34]. In addition to cytoskeletal disruption, TcdA and TcdB induce cell death through both caspase-dependent and caspase-independent pathways, further exacerbating epithelial barrier injury [35].

##### Enterotoxigenic *Bacteroides fragilis* (ETBF)

Enterotoxigenic *Bacteroides fragilis* (ETBF) carries the *bft* gene, which encodes the Bacteroides fragilis toxin (BFT). This enterotoxin has been associated with disease activation or flares in IBD [36]. Clinical studies have shown that the *bft* gene can be detected in intestinal biopsy specimens from 51.4% of patients with ulcerative colitis, compared with only 1.6% in healthy controls [37], suggesting a strong association with mucosal inflammation. Mechanistically, BFT binds to receptors on the surface of intestinal epithelial cells and cleaves the adherens junction protein E-cadherin, thereby disrupting epithelial barrier integrity [38].

##### *Campylobacter concisus* 

*Campylobacter concisus* is a facultative intracellular, invasive Gram-negative bacterium that has been repeatedly associated with IBD in clinical and microbiological studies [39,40]. Although its primary reservoir is the human oral cavity, *C. concisus* can stably colonize the intestinal mucosa, and strains of oral origin have been epidemiologically and functionally linked to intestinal barrier disruption, mucosal inflammation, and IBD pathogenesis [41]. The zonula occludens toxin (Zot), encoded by *zot* of *C. concisus*, increases intestinal epithelial barrier permeability and induces structural disassembly of tight junctions [42]. Moreover, Zot stimulates intestinal epithelial cells and macrophages to secrete pro-inflammatory cytokines, particularly TNF-α, thereby contributing to the pathogenesis and clinical relapse of IBD [43]. Furthermore, *C. concisus* contributes to epithelial sodium channel dysfunction through an IL-32-mediated ERK1/2 signaling pathway and claudin-8–dependent barrier impairment, leading to reduced Na^+^ absorption and intestinal inflammation [44]. The bacterium also promotes intestinal epithelial cell apoptosis, downregulates tight junction protein claudin-5 expression, and enhances the production of inflammatory mediators such as IL-8 and TNF-α [45].

##### *Fusobacterium nucleatum* 

A Canadian study has identified significantly higher isolation rates of *Fusobacterium nucleatum* from intestinal biopsies of IBD patients compared with healthy controls [46]. Recent investigations have confirmed increased abundance of this bacterium in intestinal tissues from UC and IBD patients, with levels positively correlating with disease severity [47,48]. In dextran sulfate sodium (DSS)-induced colitis mouse models, *F. nucleatum* induces differentiation of Th1 and Th17 cells, promotes secretion of pro-inflammatory cytokines including TNF-α, IFN-γ, IL-1β, IL-6, and IL-17, and disrupts the intestinal epithelial barrier through induction of autophagic epithelial cell death [49].

##### Adherent-Invasive *Escherichia coli* (AIEC)

Adherent-invasive *Escherichia coli* (AIEC) is enriched in the intestinal microbiota of CD patients and represents a key factor in maintaining intestinal inflammation [50]. This bacterium encodes propanediol dehydrogenase (PduC), which promotes fucose fermentation and triggers intestinal T cell inflammation [51]. In mouse models, AIEC-induced Th17 cell activation and IL-1β production depend on the response of CX3CR1^+^ mononuclear phagocytes to PduC [52]. AIEC binds to carcinoembryonic antigen-related cell adhesion molecule 6 (CEACAM6) on the surface of intestinal epithelial cells and macrophages through the FimH adhesin at the tip of type 1 pili [53]. Following internalization, AIEC replicates within phagosomes and evades immune clearance, while disrupting tight junction proteins and increasing intestinal permeability [54]. In genetically susceptible individuals (with *NOD2*, *ATG16L1*, or *IRGM* mutations), AIEC induces sustained TNF-α activation and strongly drives Th1/Th17 immune responses, thereby establishing a self-amplifying cycle of ‘invasion–survival–inflammation’ that results in dysregulated intestinal immunity and chronic disease progression [55,56].

#### 2.1.2. Other Eukaryotic Microorganisms

Recent studies have demonstrated that microsporidia infection significantly exacerbates colonic pathology in the DSS-induced murine model of IBD [57]. Mechanistically, microsporidia compromise intestinal barrier function through three interrelated pathways: (i) increasing epithelial paracellular permeability, (ii) impairing epithelial restitution and wound healing, and (iii) inducing disassembly and downregulation of the tight junction protein ZO-1 [57]. These findings provide direct experimental evidence that certain IBD-associated eukaryotic microbes, notably microsporidia, can actively drive disease progression by eroding intestinal barrier integrity. Notably, dysbiotic shifts in the gut microbiota, including both bacterial and fungal components, are increasingly recognized as key contributors to chronic intestinal inflammation and barrier dysfunction in IBD.

#### 2.1.3. Viral Pathogens

In the IL-10^−/−^ mouse model of IBD, murine norovirus (MNV) infection triggers intestinal pathology characterized by transcriptional downregulation of key tight junction genes and increased epithelial apoptosis [58]. Specifically, MNV infection significantly reduces mRNA and protein expression of Claudin-4, Claudin-8, and occludin in both the colon and small intestine of IL-10^−/−^ mice [58]. These findings demonstrate that enteric viral infection, particularly in the context of defective IL-10-mediated immune regulation, can actively disrupt epithelial barrier integrity, thereby contributing to IBD pathogenesis rather than merely acting as a passive bystander.

### 2.2. Gut Microbiota Dysbiosis

#### 2.2.1. Characteristics of Gut Microbiota Dysbiosis in IBD

The human intestinal microbiota is a highly structured, multi-kingdom ecosystem comprising bacteria, fungi and viruses [59]. Bacteria remain the most extensively characterized component, owing to their numerical dominance and well-documented functional roles in host physiology. In health, Firmicutes, encompassing commensal genera such as *Lactobacillus*, *Ruminococcus*, *Clostridium*, *Enterococcus*, and *Bacillus*, predominate the bacterial community, followed by Bacteroidetes, primarily represented by *Bacteroides* and *Prevotella* species [60]. Notably, the class Clostridia constitutes approximately 95% of all Firmicutes, underscoring its centrality to the phylum’s ecological and metabolic contributions [61]. By contrast, the intestinal mycobiota exhibits markedly lower taxonomic diversity: fewer than 20 fungal species are typically detected in healthy individuals, with pronounced inter-individual variation in composition and abundance [62]. Dominant colonizers include *Candida*, *Saccharomyces*, *Aspergillus*, *Malassezia*, and *Penicillium* [63]. The gut virobiota is overwhelmingly dominated by temperate bacteriophages, particularly members of the order Caudovirales (including Podoviridae, Siphoviridae, and Myoviridae) and Microviridae; Inoviridae are less prevalent in the gut compared to other niches. While phage populations display substantial interpersonal heterogeneity, they exhibit remarkable intra-individual stability over time [64,65], suggesting active host- and microbiota-mediated regulation. Collectively, these finely tuned, multi-kingdom microbe–host interactions constitute the biological foundation of intestinal homeostasis; disruption of this balanced symbiosis defines dysbiosis in its broadest sense, namely, a state of homeostatic failure [66,67].

Accumulating evidence demonstrates that patients with IBD exhibit taxon-specific bacterial dysbiosis: consistent reductions in beneficial Firmicutes (including *Roseburia* and *F. prausnitzii*), *Bacteroides* species, and other anti-inflammatory commensals, alongside expansions of pro-inflammatory Proteobacteria, certain Bacteroidetes lineages, and mucosa-associated bacteria, including *Ruminococcus gnavus* and *Ruminococcus torques* [68,69]. Critically, elevated serum titers of anti-*Saccharomyces cerevisiae* antibodies (ASCA) are a well-established serological hallmark of CD [70,71], reflecting loss of immune tolerance to fungal antigens and implicating mycobiota-directed immunity in disease pathogenesis. Furthermore, genetic susceptibility loci for IBD, including CLEC7A (encoding Dectin-1) and CARD9, encode key components of the antifungal innate immune pathway [72], providing mechanistic evidence that fungal and bacterial dysbiosis converge on shared host immune defects. Bacterial dysbiosis may further exacerbate fungal expansion by depleting colonization-resistant niches and altering metabolite availability, thereby establishing a self-reinforcing, multi-kingdom pathogenic loop. Viral dysbiosis is equally prominent: active CD and UC patients display increased gut virome α-diversity [73]. Pediatric CD is associated with significantly elevated Caudovirales phage abundance relative to healthy controls [74], In UC, mucosa-associated viromes, dominated by Caudovirales bacteriophages, exhibit high abundance but markedly reduced α-diversity and evenness [75,76].

**Figure 1:**
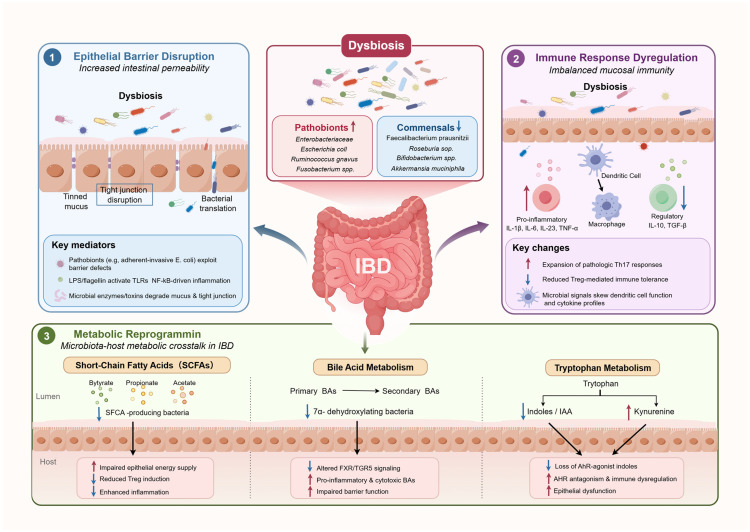
Gut Microbial Dysbiosis Drives Inflammatory Bowel Disease via Barrier, Immune, and Metabolic Dysfunction. Gut microbial dysbiosis is a key driver of IBD. It is marked by loss of beneficial commensals and expansion of pathobionts. Dysbiosis disrupts epithelial barrier integrity, by reducing mucus production, impairing tight junctions, and increasing barrier permeability. These changes lead to microbial translocation and immune activation. Dysbiosis also triggers immune dysregulation. This occurs via PRRs (TLRs, NLRs, CLRs). PRRs activate NF-κB, JAK/STAT, NLRP3 inflammasome, and IL-23/Th17 pathways. These pathways promote pro-inflammatory cytokines and weaken Treg-mediated tolerance. In addition, dysbiosis alters microbial metabolism, including reduced SCFAs, impaired bile acid conversion (FXR/TGR5), and disrupted tryptophan–AhR signaling, further aggravating barrier and immune dysfunction. IBD: Inflammatory Bowel Disease; BA: bile acid; IAA: Indole-3-Acetic Acid; FXR: Farnesoid X Receptor; TGR: Takeda G protein–coupled receptor 5; AhR: Aryl hydrocarbon Receptor.

Under homeostatic conditions, the intestinal commensal microbiota sustain tonic stimulation of antigen-presenting cells, including dendritic cells and macrophages, to promote regulatory T cell differentiation, enforce mucosal immune tolerance, and thereby prevent spontaneous intestinal inflammation [77]. However, in genetically susceptible hosts, dysbiotic microbial communities, particularly those enriched in pro-inflammatory Proteobacteria and select Bacteroidetes lineages, and depleted of immunomodulatory Firmicutes, can subvert this regulatory axis, triggering immune activation, loss of tolerance, and ultimately colitis. This dysbiotic signature represents a consistent microbiological hallmark of IBD [78,79]. Moreover, antibiotic exposure exacerbates IBD risk not only by depleting beneficial taxa but also by impairing microbial metabolic functions essential for epithelial barrier integrity and immune education (as shown in Figure 1), underscoring that microbial ecological stability encompasses both compositional resilience and functional redundancy [80]. Although the precise sequence of events initiating IBD remains incompletely defined, microbiota dysbiosis within the inflamed intestinal microenvironment is now widely accepted as a central, non-redundant pathological driver, not merely an epiphenomenon.

#### 2.2.2. Dysbiosis and Intestinal Barrier Dysfunction in IBD Pathogenesis

Intestinal barrier integrity is dynamically maintained through tightly regulated crosstalk between the gut microbiota and host intestinal epithelial cells. Disruption of this dialogue is a well-established pathogenic driver of IBD. Under homeostatic conditions, commensal bacteria act cooperatively to sustain barrier function, among them, *F. prausnitzii* stands out as a keystone protective species whose depletion is consistently associated with IBD onset and severity [81]. *F. prausnitzii* enforces barrier resilience via two experimentally validated mechanisms. First, its secreted microbial anti-inflammatory molecule (MAM) selectively inhibits NF-κB activation in epithelial and immune cells, thereby attenuating inflammation-driven epithelial apoptosis and tight junction disassembly [82]. Second, as a dominant colonic butyrate producer, it supplies butyrate that activates epithelial PPAR-γ signaling, upregulates claudin-1, occludin, and ZO-1 expression, and enhances mitochondrial function, collectively reinforcing epithelial barrier competence [83]. Critically, clinical cohort studies confirm that *F. prausnitzii* abundance is significantly reduced in both CD and UC patients, and this loss correlates strongly with impaired barrier function and disease activity, establishing a robust association consistent with causal contribution. Similarly, *Roseburia intestinalis* promotes barrier maturation through TLR5-dependent recognition of its flagellin by intestinal epithelial cells, leading to transcriptional upregulation of occludin and MUC2, key effectors of paracellular sealing and mucus layer formation [84]. *Akkermansia muciniphila* further fortifies the barrier via extracellular vesicle, mediated delivery of bioactive molecules that stimulate goblet cell differentiation and MUC2 synthesis, while concurrently suppressing expansion of pathobionts (including AIEC and other *Proteobacteria*) [52].

Corresponding to the reduction in protective symbiotic bacteria, the significant enrichment of Gram-negative bacteria in the intestines of IBD patients constitutes an important driver of barrier damage, especially the expansion of Enterobacteriaceae has become a hallmark of IBD microbiota dysbiosis [85]. The pathological effects of this microbiota structural change are manifested at multiple levels: as the dominant phylum of Gram-negative bacteria, the expansion of Proteobacteria continuously exposes the mucosal immune system to pathogen-associated molecular patterns (PAMPs), such as lipopolysaccharides and flagellin, triggering excessive activation of innate immunity. Specifically, it is manifested as the loss of epithelial barrier integrity, excessive production of antimicrobial peptides and pro-inflammatory cytokines, and abnormal activation of IECs and APCs, forming a vicious cycle of inflammatory cascade amplification [85]. In addition, some members of the Proteobacteria phylum directly damage the barrier structure of intestinal epithelial cells through their adhesion and invasion capabilities, alter the composition of the intestinal microbiota and induce chronic inflammatory responses, further exacerbating barrier dysfunction. Notably, *Bacteroides thetaiotaomicron*, a core symbiotic bacterium in healthy intestines, is significantly reduced in patients with CD and UC [86]. Its functional deficiency reveals the mechanism of barrier damage from another perspective. As an acetate producer, *B. thetaiotaomicron* forms a metabolic mutualism with acetate consumers and butyrate producers such as *F. prausnitzii*, jointly regulating the glycosylation modification of small intestinal mucus cells and maintaining the physicochemical properties of the mucus barrier [87]. At the same time, *B. thetaiotaomicron* can stimulate Paneth cells to release the antimicrobial peptide Ang4, strengthening the mucosal immune defense system. Its reduction leads to the dual weakening of the chemical and immune barriers [88].

Beyond bacterial imbalances, changes in the gut’s viral community, the virome, also play a subtle but meaningful role in supporting (or sometimes challenging) the health of the intestinal barrier. Research using animal models inspired by Gulf War illness has offered valuable insights [89]. When the virome becomes unbalanced, subtle yet important shifts can occur in the structures that help seal the gut lining, particularly the tight junctions. Scientists have observed a modest increase in Claudin-2 alongside a soft decline in ZO-1 and occludin [89]. These findings suggest that certain viruses may influence how these barrier gatekeepers are expressed, not as invaders, but as quiet participants in a finely tuned system. Taken together, the shifts across the entire gut microbiome reflect a broader story of balance being gently tipped: protective functions may soften, while responsive or reactive elements become more active. This delicate recalibration affects all three layers of our gut’s natural defense system: the physical seal, the chemical shield, and the immune sentinels, making it harder for the gut to return to calm, steady harmony.

#### 2.2.3. Dysbiosis and Dysregulation of Immune Responses in IBD

The genetic architecture of IBD spans a continuous spectrum, from high-penetrance familial aggregation to polygenic population-level susceptibility. Genome-wide association studies (GWAS) have now identified more than 240 independent risk loci, collectively explaining ~25% of disease heritability [90]. Cross-population genetic comparisons offer critical insights into disease heterogeneity. A landmark GWAS in East Asian cohorts identified 80 IBD-associated loci, and meta-analysis integrating these with data from over 30,000 European cases uncovered 81 novel, trans-ancestral risk loci, many mapping to immune-relevant enhancers and non-coding regulatory elements [91]. Strikingly, while the overall burden of common risk variants is comparable between East Asian and European populations, CD exhibits significantly higher Single Nucleotide Polymorphism (SNP)-based heritability than UC, revealing fundamental differences in genetic architecture [91]. This divergence likely reflects CD’s stronger enrichment for variants disrupting innate immune sensing (e.g., *NOD2*, *CARD9*, *ATG16L1*) and autophagy, whereas UC shows greater sensitivity to environmental modifiers. Beyond common SNPs, the genetic landscape of IBD expands hierarchically across variant classes. Rare, large-effect structural variants, including chromosomal aneuploidies and copy number variations, perturb immune homeostasis via dosage-sensitive mechanisms. For example, recurrent 16p11.2 deletions reduce expression of the immunoregulatory gene CORO1A, while duplications at 1q21.1 amplify pro-inflammatory cytokine genes, collectively contributing to early-onset, treatment-refractory disease and familial clustering [92]. At the ultra-rare coding variant level, deep whole-exome sequencing of >30,000 IBD patients identified statistically robust, loss-of-function associations for ATG4C and PDLIM5, directly implicating defective autophagy–lysosomal degradation and dysregulated STAT3 nuclear shuttling in CD pathogenesis, thereby bridging monogenic-like mechanisms with complex disease biology [93]. Collectively, these genetic discoveries converge on a limited set of core molecular signaling pathways—most notably NF-κB, JAK/STAT, NLRP3 inflammasome, and IL-23/Th17 axis—that serve as critical nodes linking genetic predisposition to immune dysregulation in IBD.

Intestinal intraepithelial lymphocytes, often thought of as the gut’s gentle first responders, are among the very first immune cells to greet microbes as they pass through the intestinal lining. Remarkably, certain friendly residents of our gut, like segmented filamentous bacteria (SFB), help coach these lymphocytes during early life and throughout adulthood, supporting their balanced activation and helping them distinguish between harmless neighbors and genuine threats [77]. This quiet, ongoing training is one of the many ways our microbiome contributes to steady, calm mucosal immunity. But when microbial balance is disturbed, this finely tuned dialogue can soften or shift, making it harder for the immune system to stay centered. For example, in some people with IBD, fungi like *Candida albicans* may become more prominent, gently encouraging the body to produce slightly higher levels of signaling molecules such as TNF-α, IL-17A, and IFN-γ [94]. This is not due to invasion or aggression, it reflects a subtle recalibration of metabolic pathways (like glutamine handling) guided by the dectin-1-Syk-NF-κB axis, which helps shape a local environment where immune activity is naturally elevated. Similarly, certain bacterial groupsm, including *Oscillibacter* in UC patients, tend to appear alongside modest increases in IL-6 and IL-1β, and correlate with how people feel [95]. Likewise, *Ruminiclostridium* has been observed to rise alongside similar signals in experimental models of gut inflammation [96]. These patterns remind us that microbes do not act in isolation, for they are part of a living conversation with our immune system. Even the gut’s viral community plays a supportive role. Studies show that phages help maintain balance by guiding bacterial populations. When this balance shifts, as seen in UC, phage activity can subtly influence IFN-γ levels, not as a trigger of harm, but as part of a broader immune conversation [76,97]. Importantly, research in mice shows that preserving a healthy, diverse virome helps strengthen resilience against gut inflammation, and this protection relies partly on receptors like TLR3 and TLR7, which recognize viral signals not to sound an alarm, but to quietly support protective immunity [97]. In short, every member of our gut ecosystem, from bacteria and fungi to viruses, has a voice in the ongoing, compassionate dialogue that keeps our gut healthy.

The molecular basis of this host–microbiota dialogue is governed by a conserved system of pattern recognition receptors (PRRs) that convert microbial signals into host immune responses. Toll-like receptors (TLRs), NOD-like receptors (NLRs), and C-type lectin receptors (CLRs) serve as primary sensors of microbial structural components—lipopolysaccharide, flagellin, peptidoglycan, and β-glucan—initiating downstream inflammatory cascades upon activation [98,99]. Genetic susceptibility in IBD converges on these sensing and signaling nodes: risk variants in *NOD2*, *CARD9*, and *ATG16L1* impair microbial clearance and antigen presentation, while IL23R variants modulate the threshold for Th17 polarization [90,91,92,93]. Once activated, these pathways coalesce into four core signaling axes—NF-κB, JAK/STAT, NLRP3 inflammasome, and IL-23/Th17—that together orchestrate the transition from microbial imbalance to sustained intestinal inflammation [100]. NF-κB serves as a master transcriptional regulator of pro-inflammatory cytokines (TNF-α, IL-6, IL-1β), while JAK/STAT signaling, particularly STAT3, drives Th17 differentiation and suppresses Treg stability [100,101]. The NLRP3 inflammasome amplifies inflammation through IL-1β and IL-18 maturation, and the IL-23/Th17 axis stabilizes pathogenic T-cell responses [102]. Critically, these pathways do not operate in isolation but form an interconnected network that locks the mucosal immune system into a self-perpetuating state of activation, wherein microbial dysbiosis, barrier disruption, and immune dysregulation reinforce one another.

In IBD, intestinal immune regulation is profoundly impaired, resulting in sustained inflammation and a failure to constrain pathogenic T-cell responses. While Th1- and Th17-polarized immunity normally serves protective antimicrobial functions, their dysregulated activation drives epithelial barrier disruption, crypt abscess formation, and transmural tissue injury, hallmarks of IBD pathology [103]. Central to this pathogenic T-cell response is the IL-23/Th17 axis, wherein IL-23 stabilizes pathogenic Th17 cells and sustains IL-17A production, thereby amplifying mucosal inflammation [104]. Foxp3^+^ regulatory T cells (Tregs) are indispensable for maintaining mucosal tolerance. Functional impairment or numerical deficiency of colonic Tregs is causally linked to chronic intestinal inflammation and disease progression in both human IBD and experimental colitis models [105]. Critically, this Treg dysfunction is exacerbated by depletion or functional compromise of immunomodulatory commensal bacteria, a key contributor to the breakdown of oral and mucosal tolerance [106,107].

Cross-species functional validation bridges human genetic associations with causal disease mechanisms. Integrated transcriptomic profiling across 13 preclinical IBD mouse models and matched human intestinal biopsies from >2500 IBD patients identified 283 evolutionarily conserved, functionally active GWAS risk loci, encompassing key regulatory nodes in T cell homing, innate immune activation (e.g., *NOS2*, *LCN2*, *IL6*, *OSM*), and epithelial barrier integrity (e.g., *ABCB1*, *SLC4A4*, *PCK1*, *AQP8*) [108]. This cross-model conservation robustly validates the physiological relevance of murine systems for IBD drug discovery and prioritizes JAK-STAT signaling, Th17 differentiation, and NF-κB activation as high-confidence, therapeutically tractable pathways. Indeed, these three pathways—JAK/STAT, NF-κB, and IL-23/Th17—form an interconnected signaling network that drives cytokine production, T-cell polarization, and sustained inflammation in the IBD gut [109]. *F. prausnitziI*, a keystone symbiont consistently depleted in IBD, orchestrates multi-layered immunoregulation: its culture supernatant potently inhibits NF-κB nuclear translocation and IL-8 secretion in human intestinal epithelial Caco-2 cells [110], confirming that soluble mediators, not merely live bacteria, are sufficient for anti-inflammatory activity. Mechanistically, *F. prausnitzii*-derived butyrate inhibits histone deacetylases (HDACs) to promote Foxp3 expression and Treg differentiation. Concurrently, it accelerates c-Myc protein degradation and disrupts the IL-6/STAT3/IL-17 signaling axis, thereby suppressing Th17 lineage commitment [111]. Its secreted 15 kDa MAM directly inhibits NF-κB–driven colitis and restores transepithelial electrical resistance [112]. Moreover, *F. prausnitzii* enhances the frequency and suppressive capacity of CD25^+^Foxp3^+^ Tregs in human peripheral blood mononuclear cells (PBMCs) and murine splenocytes, while its extracellular polymeric substances induce dendritic cell, thereby reinforcing Treg stability and function [113]. *Roseburia intestinalis* complements this regulation via TLR5-dependent induction of thymic stromal lymphopoietin (TSLP) in intestinal epithelial cells, which licenses DC to produce IL-10 and TGF-β, cytokines essential for peripheral Treg induction [114]. Notably, *R. intestinalis* and its butyrate metabolite synergistically upregulate epithelial TLR5 expression, amplifying flagellin–TLR5 signaling and downstream anti-inflammatory cytokine output [115]. Non-toxigenic *Bacteroides fragilis* (NTBF) exerts strain-specific immunoregulation through polysaccharide A (PSA), which signals via TLR2 on CD4^+^ T cells to drive Foxp3^+^ Treg differentiation and suppress IL-17/TNF-α production while enhancing colonic IL-10 synthesis, conferring robust protection against TNBS-induced colitis [116]. PSA-derived butyrate further inhibits M1 macrophage polarization by suppressing NF-κB and HIF-1α activity, reducing nitric oxide (NO) and IL-12 secretion [117]. *B. thetaiotaomicron*, the second most abundant Bacteroides species in healthy human microbiomes, exhibits marked depletion in CD and UC [87]. Functionally, *B. thetaiotaomicron* promotes Treg and Th2 differentiation while suppressing Th1/Th17 polarization, reflected in elevated colonic IL-10 and diminished IFN-γ/IL-17 levels in gnotobiotic models [118]. In contrast, pathobionts such as *Fusobacterium varium* exploit host vulnerabilities. Its preferential adhesion to and invasion of intestinal epithelial cells triggers robust IL-8 and TNF-α release, initiating neutrophil recruitment and epithelial damage [119]. Genomic analyses confirm that F. varium encodes a type V secretion system (T5SS) and the adhesin/invasin FadA, both required for mucosal adherence, epithelial internalization, and NLRP3 inflammasome activation [120]. This NLRP3 inflammasome pathway represents a critical node where microbial signals converge to drive IL-1β and IL-18 maturation, thereby amplifying the inflammatory cascade in IBD [121]. Mechanistically, a macrophage-specific enhancer within the 21q22 gene desert drives inflammatory macrophage polarization by amplifying ETS2 transcription factor expression. Pharmacologic inhibition of its downstream MEK1/2–ERK axis suppresses TNF-α and IL-23 production in human CD-derived macrophages and ameliorates colitis in vivo [122]. Complementing this, a splicing quantitative trait locus in the SBNO2 gene on chromosome 5 regulates the expression ratio of isoforms, thereby impairing macrophage bacterial killing and directly linking non-coding CD risk variants to defective innate immunity [123]. At the epigenetic level, CD-specific hypomethylation at CpG sites in the promoters of MHC-I genes and NLRC5 leads to transcriptional activation and upregulated surface MHC-I expression on intestinal epithelial cells, triggering aberrant CD8^+^ T cell activation and cytotoxicity [124].

Collectively, microbiota dysbiosis fuels immune instability not through isolated defects, but via an integrated network: bacteriophage-mediated lysis of beneficial bacteria, expansion of virulent pathobionts, and erosion of symbiont-derived immunoregulatory signals converge to collapse mucosal tolerance, culminating in self-perpetuating inflammation and irreversible tissue remodeling in IBD. Underpinning this process are four core molecular signaling pathways—NF-κB, JAK/STAT, NLRP3 inflammasome, and IL-23/Th17 axis—that together orchestrate the transition from microbial imbalance to sustained intestinal inflammation. These pathways represent not only central drivers of disease pathogenesis but also high-priority therapeutic targets, with JAK inhibitors already in clinical use and ongoing efforts targeting IL-23, NF-κB, and NLRP3 holding promise for more precise immunomodulation in IBD.

#### 2.2.4. Dysbiosis–Driven Metabolic Reprogramming in IBD

In IBD, microbial dysbiosis is not only characterized by compositional alterations of the gut microbiota, but more critically by the depletion of key microbial taxa with essential metabolic functions. This loss drives extensive metabolic reprogramming and mechanistically contributes to disease initiation and progression. Mounting evidence demonstrates that IBD patients exhibit systemic perturbations in their metabolic profiles, encompassing significantly reduced levels of medium-chain fatty acids and short-chain fatty acids (SCFAs) in fecal samples [125,126,127], disrupted bile acid (BA) metabolism [128,129], and aberrant changes in salicylic acid [130] and amino acid levels [128,131].

Compared with healthy individuals, IBD patients display markedly diminished microbial-derived anti-inflammatory metabolites [125,132], whereas certain pro-inflammatory-associated metabolites show relative enrichment [133]. These metabolic perturbations are closely linked to IBD-related shifts in microbial composition, particularly the depletion of microbial functional genes and associated enzymatic activities implicated in critical metabolic processes, resulting in the disruption of multiple core metabolic axes—principally SCFA production, BA transformation, and tryptophan (Trp) metabolic pathways. A study by Morgan et al. revealed that approximately 12% of microbial metabolic pathways were significantly altered in IBD patients relative to healthy controls [134]. Furthermore, in CD patients, the abundance of metabolic genes associated with butyrate and propionate synthesis was markedly decreased, underscoring the tight interplay between microbial functional loss and metabolic dysregulation.

##### Short-Chain Fatty Acids (SCFAs)

Among microbial metabolites, SCFAs, primarily acetate, propionate and butyrate, arise from anaerobic fermentation of dietary fiber by commensal bacteria [135]. SCFAs play pivotal roles in maintaining intestinal immune homeostasis, promoting the differentiation and expansion of colonic regulatory T cells (Tregs) [136], and suppressing the expression of multiple pro-inflammatory cytokines (such as IL-6 and IL-12) [137], thereby exhibiting marked anti-inflammatory effects. These functions are partially mediated through their binding to and activation of G protein-coupled receptors, including GPR43, expressed on the surface of colonic epithelial cells and immune cells [138,139]. In DSS-induced colitis models, GPR43 deficiency exacerbates inflammatory responses and leads to refractory colitis [140], further supporting the protective role of SCFAs in regulating intestinal inflammation.

In IBD patients, SCFA levels are consistently and significantly reduced, with butyrate exhibiting the most pronounced and sustained depletion across disease subtypes [78]. This metabolic alteration is mechanistically attributed to the depletion of obligate anaerobic bacteria encoding key enzymes for carbohydrate fermentation. Notably, major butyrate-producing species, including *Faecalibacterium prausnitzii* and *Roseburia intestinalis*, are markedly reduced in the gut microbiota of IBD patients [141,142], resulting in a global collapse of colonic fermentation capacity and resulting in insufficient butyrate production as a central metabolic defect. This metabolic deficiency carries direct functional consequences: diminished butyrate impairs mitochondrial β-oxidation capacity in colonic epithelial cells, disrupts hypoxia-inducible factor-1α (HIF-1α)-dependent barrier homeostasis programs, consequently leading to tight junction structural compromise and compromised barrier function [143,144]. Concurrently, butyrate deficiency attenuates its inhibitory effects on histone deacetylases (HDACs), relieving the negative regulation of pro-inflammatory transcriptional pathways such as NF-κB and amplifying inflammatory responses [110,111,112,145]. These multifaceted mechanisms collectively promote intestinal barrier disruption and perpetuation of chronic mucosal inflammation. Importantly, Butyrate supplementation has demonstrated therapeutic benefits in UC patients [146], further supporting that the depletion of butyrate-producing bacteria and declining butyrate levels constitute a critical metabolic foundation underlying the pathogenesis and progression of IBD.

##### Bile Acid (BA)

In BA metabolism, IBD similarly manifests functionally relevant disturbances that are closely linked to gut dysbiosis and supported by well-defined mechanistic underpinnings. Multiple studies have reported significant alterations in fecal bile acid profiles among IBD patients [147,148]. In health, commensal bacteria express bile salt hydrolases (BSHs) and 7α-dehydroxylase enzymes that transform hepatic primary bile acids (PBAs) into a structurally diverse pool of over twenty secondary bile acids (SBAs) [149]. This microbial biotransformation establishes a dynamic BA signaling network that regulates host metabolism, barrier integrity, and immune homeostasis. However, in IBD patients, fecal and serum levels of PBAs (including cholic acid, glycocholic acid, and taurocholic acid) are significantly elevated, whereas SBAs (especially lithocholic acid and deoxycholic acid) are markedly depleted [78]. This imbalance is mechanistically linked to the selective loss of BA-transforming taxa, notably Blautia obeum, Anaerobutyricum hallii, and Eubacterium rectale, species experimentally validated to harbor functional BSH and 7α-dehydroxylase activity [128,150]. Critically, SBAs, such as lithocholic acid and deoxycholic acid, are not mere metabolic byproducts but potent endogenous signaling molecules, that function as ligands for the G protein–coupled receptor TGR5, thereby actively participating in host signaling regulation.

A reduction in SBAs leads to attenuated TGR5 signaling, which in turn disrupts epithelial regeneration processes mediated by the SRC/YAP pathway and weakens anti-inflammatory signaling in lamina propria macrophages, thereby impairing the suppression of the NLRP3 inflammasome [151,152]. Clinically, CD patients whose fecal BA profiles are dominated by PBAs exhibit significantly shorter remission durations and higher rates of endoscopic recurrence following surgery or biologic therapy, underscoring the critical pathogenic significance of microbiota-driven bile acid metabolic dysregulation in disease initiation and progression [153].

##### Tryptophan (Trp)

Dysregulation of the Trp metabolism constitutes a third critical mechanistic axis linking microbial dysbiosis to immune dysfunction. In a large cohort study encompassing 535 patients, Nikolaus et al. demonstrated that circulating Trp levels were significantly negatively correlated with disease activity, indicating Trp metabolic disturbance in inflammatory progression [154]. Mechanistically, this metabolic aberration directly stems from the depletion of Trp-metabolizing functional microbial communities [155,156]. Under healthy conditions, commensal gut bacteria convert Trp into diverse bioactive indole derivatives via the indole pathway, whereas in IBD, the reduction in relevant bacterial populations limits the generation of these metabolites, resulting in the loss of critical immunoregulatory signals.

Crucially, Trp metabolites act not only as metabolic intermediates but as potent immunomodulators, regulating innate and adaptive immune responses by activating the aryl hydrocarbon receptor (AHR) [145,155,157]. AHR expression is significantly downregulated in inflamed ileal and colonic tissues from CD patients versus non-inflamed controls [158], and pharmacologic AHR activation by indole-3-propionic acid restores mucosal homeostasis in DSS-induced colitis through canonical AHR-IL-22 signaling [158]. Correspondingly, Lactobacillus strains capable of producing aryl AHR agonists have been shown to significantly ameliorate DSS-induced colitis [159], further supporting the functional importance of the microbiota–Trp–AHR axis. These convergent lines of evidence position the microbiota–Trp–AHR axis as a high-priority, mechanistically grounded therapeutic target for restoring immune–epithelial crosstalk in IBD.

Beyond AHR, Trp metabolites also participate in barrier regulation through activation of the pregnane X receptor (PXR). Indole-3-propionic acid (IPA) serves as a PXR ligand to suppress TNF production and enhance intestinal epithelial barrier function in animal models [160,161]. Notably, IPA levels are significantly reduced in UC patients [156], further indicating the compromised state of this pathway in disease. Collectively, these findings establish tryptophan metabolic dysregulation as a crucial bridge connecting microbial functional impairment to dysregulated immune–epithelial interactions.

### 2.3. Gut-Brain Axis

#### 2.3.1. IBD, Psychological Stress, and the Rationale for a Gut–Brain Axis Framework

IBD is increasingly recognized not merely as a localized intestinal disorder, but rather as a systemic immune-mediated condition characterized by complex interplay among environmental, immune, and neuroendocrine factors [162]. A robust and bidirectional association between IBD and psychiatric comorbidities—including stress, anxiety, and major depressive disorder—have been consistently demonstrated [163]. Psychological stress exerts direct physiological effects on the gut: it increases intestinal permeability, alters colonic motility and visceral sensation, modulates epithelial secretion, and induces compositional and functional alterations in the gut microbiota, thereby promoting colitis initiation and reactivation in murine models [164].

From a clinical perspective, heightened psychological stress represents not merely a consequence, but an independent predictor of IBD relapse, hospitalization, and therapeutic non-response [165]. Critically, early life stress is associated with a 2- to 3-fold increased risk of subsequent IBD development [166]. Conversely, IBD itself confers substantial burden of psychiatric morbidity, with significantly increased risks of anxiety (HR 1.48) and depression (HR 1.55) following diagnosis [167]. Disease activity further correlates with psychological burden, as patients with active IBD exhibit markedly higher rates of anxiety and depression compared with those in remission [168].

**Figure 2:**
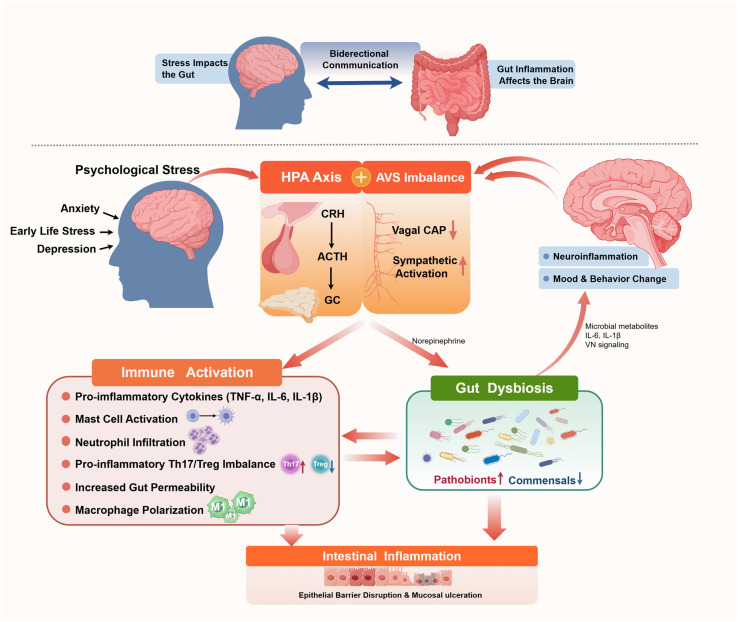
Gut-Brain Axis-Mediated Pathogenesis of Inflammatory Bowel Disease. Psychological stress activates central nervous system (CNS) pathways, thereby engaging both the hypothalamic–pituitary–adrenal (HPA) axis and the autonomic nervous system (ANS), leading to neuroendocrine and neural dysregulation. Activation of the HPA axis induces corticotropin-releasing hormone (CRH) and glucocorticoid signaling, which promote intestinal barrier disruption and pro-inflammatory immune responses. Concurrently, ANS imbalance—characterized by reduced vagal tone and enhanced sympathetic activity—further amplifies inflammation through cholinergic and adrenergbasic pathways. These signals converge on the gut microbiota, driving dysbiosis and reshaping host–microbial interactions. At the immune level, the gut–brain axis promotes Th17/Treg imbalance, macrophage polarization, and neutrophil recruitment, collectively resulting in mucosal immune dysregulation. Barrier disruption facilitates antigen translocation and sustains chronic intestinal inflammation. Importantly, peripheral inflammatory signals feed back to the CNS via circulating cytokines and neural pathways, establishing a self-reinforcing neuroimmune loop that perpetuates disease progression. HPA: hypothalamic–pituitary–adrenal axis; ANS: autonomic nervous system; CRH: corticotropin-releasing hormone; ACTH: adrenocorticotropic hormone; GC: glucocorticoids; CAP: cholinergic anti-inflammatory pathway; VN: vagus nerve.

These clinical and experimental observations indicate that CNS (CNS)-mediated processes are not simply secondary consequences of chronic inflammation, but active participants in disease modulation. This has facilitated conceptualization of the GBA, a bidirectional communication network integrating neural, endocrine, immune, and microbial signaling pathways (as shown in Figure 2) [169,170]. Through these interconnected pathways, psychological stress can disrupt intestinal homeostasis, while intestinal inflammation, in turn, influences brain function and behavior.

#### 2.3.2. Neuroendocrine Regulation: HPA Axis Activation in IBD

The hypothalamic–pituitary–adrenal (HPA) axis represents the principal neuroendocrine pathway linking psychological stress to intestinal inflammation [164,171]. Acute stress triggers the release of corticotropin-releasing hormone (CRH) from the paraventricular nucleus of the hypothalamus, thereby activating the pituitary-adrenal cascade and elevating systemic glucocorticoid levels. However, beyond its classical anti-inflammatory actions, CRH exerts direct pro-inflammatory effects within the gut. It promotes mast cell degranulation, enhances Th1/Th17 polarization, disrupts epithelial tight junction integrity, and increases production of TNF-α, IL-6, and IL-1β [172,173]. Human studies provide robust translational support: psychosocial stress significantly increases intestinal permeability in high cortisol responders [174], and exogenous CRH administration can replicate this effect, which is abolished by mast cell stabilization, thereby identifying mast cells as critical downstream effectors [175,176]. Under chronic stress conditions, sustained glucocorticoid signaling induces glucocorticoid receptor-dependent transcriptional reprogramming, leading to suppression of barrier-protective proteins (e.g., occludin and claudin-1) alongside upregulation of the pore-forming protein claudin-2. This results in a selective “leaky gut” phenotype that facilitates luminal antigen translocation [177,178].

Importantly, CRH also amplifies intestinal inflammation through immune cell reprogramming. In particular, CRH promotes functional reprogramming of macrophages toward a pro-inflammatory phenotype, characterized by enhanced inflammatory signaling and increased susceptibility to tissue injury [179,180]. Mechanistically, this process is tightly linked to autophagy-related pathways involving ATG5 and ATG7, which amplify inflammatory responses under stress conditions [181]. Experimental evidence has demonstrated that inhibition of autophagy attenuates CRH-induced colitis severity, whereas its activation exacerbates cytokine production and epithelial damage, highlighting the functional importance of the CRH–autophagy axis in stress-related intestinal inflammation [182,183], highlighting the functional importance of the CRH–autophagy axis in stress-related intestinal inflammation.

#### 2.3.3. Neural Pathways: Autonomic Nervous System Dysregulation in IBD

The autonomic nervous system (ANS) constitutes a central neural branch of the GBA, regulating intestinal homeostasis through dynamic equilibrium between parasympathetic (vagal) and sympathetic activities. In IBD, this balance is disrupted, manifesting as diminished vagal tone and enhanced sympathetic activation [183,184].

The vagus nerve exerts potent anti-inflammatory effects via the cholinergic an-ti-inflammatory pathway (CAP) by activating α7 nicotinic acetylcholine receptors (α7nAChR) on macrophages via acetylcholine release, thereby suppressing NF-κB signaling and cytokine production [185,186,187,188]. Both clinical and preclinical evidence support its protective role: vagal nerve stimulation attenuates colitis severity, whereas reduced vagal rhythmicity in patients correlates with elevated inflammatory cytokine levels [189,190]. Notably, vagotomy is associated with significantly increased IBD risk, further supporting the pathogenic contribution of vagal dysfunction [191]. Conversely, sympathetic activation promotes inflammation through catecholaminergic signaling. Stress-induced adrenergic pathway activation enhances neutrophil infiltration, epithelial damage, and cytokine production via α- and β-adrenergic receptors [192,193,194]. Furthermore, sympathetic signaling directly targets intestinal epithelial cells through the β-AR-DUOX2-NADPH oxidase axis [194], increasing oxidative stress and compromising barrier integrity, thereby exacerbating IBD. Importantly, emerging evidence indicates that the enteric nervous system (ENS) serves as a critical intermediary, translating central autonomic signals into local intestinal immune responses [195]. Under psychological stress conditions, autonomic inputs induce structural and functional remodeling of the ENS, including increased enteric plexus density and expansion of cholinergic neuronal, which are associated with enhanced epithelial permeability and barrier dysfunction [196,197].

Thus, psychological stress induces coordinated autonomic alterations—simultaneous suppression of parasympathetic activity and activation of sympathetic output—thereby synergistically amplifying both systemic and intestinal inflammation [198,199]. This dual autonomic dysregulation drives concerted upregulation of IL-1β, IL-6, and TNF-α in both central and peripheral compartments, as supported by a high-quality meta-analysis of 42 clinical studies. Importantly, restoration of vagal tone through transcutaneous vagal nerve stimulation has demonstrated clinical efficacy in reducing both psychological and somatic symptoms and intestinal inflammation in CD [200,201], underscoring the therapeutic potential of targeting neuroimmune circuits within the GBA.

#### 2.3.4. Immune Mechanisms of the Gut–Brain Axis in IBD

At the immune level, the GBA orchestrates intestinal inflammation through tightly integrated regulation of both adaptive and innate immune compartments, acting as a central effector arm linking neuroendocrine signaling to mucosal immune dysregulation in IBD.

A central feature of GBA-mediated immune disruption is the shift in Th17/Treg balance toward pro-inflammatory phenotypes, which intersects and synergistically amplifies with the aforementioned immune consequences of gut dysbiosis. Both experimental models and human IBD tissues consistently demonstrate increased Th17 differentiation alongside impaired Treg function [202,203]. However, in contrast to the direct microbial immunomodulation, the GBA primarily amplifies this imbalance through neuroendocrine signaling. Psycho-logical stress enhances this disequilibrium through neuroendocrine mediators: glucocorticoids and stress-associated factors suppress TGF-β and IL-2 signaling pathways, thereby impairing Treg differentiation while promoting Th17 polarization [204,205]. Concurrently, clinical data indicate that stress further enhances IL-23/IL-23R signaling [104], reinforcing the pathogenic inflammatory functions of Th17 cells on top of microbiota-driven immune activation.

Importantly, Treg dysfunction in IBD is predominantly functional rather than numerical. Stress-induced prolactin signaling reprograms dendritic cells, leading to NF-κB activation and increased production of IL-6 and IL-23, which in turn drives the conversion of Foxp3^+^ Tregs into pro-inflammatory ex-Tregs expressing IL-17 and TNF-α [206]. This neuroendocrine-driven Treg plasticity represents a critical mechanism directly linking CNS activity to the loss of intestinal immune tolerance, distinguishing it from microbiota-mediated Treg stabilization (e.g., via butyrate and microbial anti-inflammatory molecules produced by commensals such as *Faecalibacterium prausnitzii*).

Innate immune cells serve as critical intermediaries, translating signals from the CNS into localized inflammatory responses. Dendritic cells function as central sensors of neuroendocrine signals, integrating hormonal inputs (e.g., prolactin, glucocorticoids) and shaping downstream T cell responses via IL-6, IL-12, and IL-23, thereby amplifying Th17-driven inflammation [206]. Neutrophils act as rapid-response effector cells in GBA-mediated inflammation. Chronic stress activates sympathetic signaling, leading to norepinephrine-dependent upregulation of chemokines such as CXCL1 and CXCL2, which drive neutrophil mobilization and infiltration into the intestinal mucosa [193]. Once recruited, stress-induced neutrophils exhibit enhanced degranulation, increased reactive oxygen species (ROS) production, and elevated release of pro-inflammatory cytokines (IL-1β, IL-6, IL-17A), thereby contributing to epithelial damage, barrier disruption, and amplification of inflammatory cascades. Importantly, macrophage polarization induced by stress-related endocrine pathways has been comprehensively described in Section 2.3.2 and is therefore not reiterated here [179,180,181,182,183].

Innate lymphoid cells, particularly ILC3, represent a critical interface between neural signals and epithelial defense. Under physiological conditions, intestinal stromal cells support ILC3 function through neurotrophic factors, promoting IL-22 production and maintaining epithelial barrier integrity [207]. However, chronic stress-induced elevations in glucocorticoids suppresses this neuro-glial-ILC3 axis, leading to reduced IL-22 secretion and impaired mucosal defense mechanisms [208]. This increases susceptibility to microbial invasion and sustained inflammation.

Beyond individual cell types, the GBA also regulates immune responses through coordinated signaling networks. Activation of the sympathetic nervous system and the HPA axis converges on key inflammatory pathways, including NF-κB and STAT3, resulting in upregulation of IL-1β, IL-6, and TNF-α in both systemic and mucosal compartments [196,197]. Importantly, immune signaling is not unidirectional. Peripheral inflammation feeds back to the CNS through circulating cytokines and neural pathways, altering stress responsiveness and behavior, thereby establishing a self-reinforcing neuroimmune loop that sustains chronic inflammation [209]. Collectively, these findings demonstrate that the GBA orchestrates a multi-layered immune dysregulation network that directly contributes to IBD pathogenesis.

#### 2.3.5. Microbiota–Gut–Brain Axis: Mechanisms and Bidirectional Interactions in IBD

Accumulating evidence supports a mechanistically grounded bidirectional interaction between psychological stress and gut microbiota [168,210,211], rather than a purely associative relationship.

Stress-induced release of glucocorticoids and catecholamines, including norepinephrine, alters intestinal motility, mucus secretion, epithelial permeability, and luminal nutrient availability. Disruption of GBA homeostasis can thereby reshape the mucosal habitat and influence microbial ecology [183], creating a selective niche that favors pathobiont expansion while reducing commensal diversity. At the molecular level, catecholamines such as norepinephrine can directly modulate bacterial growth, virulence gene expression, and biofilm formation, promoting the expansion of Enterobacteriaceae and other inflammation-associated taxa [212,213]. Concurrently, glucocorticoid-mediated suppression of mucosal immunity impairs IgA secretion and antimicrobial peptide production, further destabilizing microbial homeostasis. These neuroendocrine-driven alterations give rise to a reproducible dysbiotic signature observed across multiple stress paradigms—including maternal separation, chronic restraint stress, and social disruption—suggesting a conserved stress–microbiota response axis [214].

Conversely, the gut microbiota actively modulates CNS function through multiple mechanistic pathways. SCFAs, including acetate, propionate, and butyrate, regulate blood–brain barrier (BBB) integrity, microglial maturation, and neuroinflammatory responses [215,216]. In germ-free (GF) mice, microglia exhibit impaired immune responses following intracerebral lipopolysaccharide (LPS) challenge; this defect can be restored by microbial colonization or SCFA supplementation [217]. Consistently, GF mice display increased BBB permeability, which is significantly reduced following microbiota reconstitution or SCFA administration [218]. In parallel, microbiota-driven tryptophan metabolism generates bioactive metabolites (e.g., indole derivatives and kynurenine pathway intermediates) that regulate serotonin biosynthesis and CNS immune signaling, thereby influencing mood and stress responsiveness [219,220]. Microbial signals are also transmitted to the brain via neural pathways. The vagus nerve serves as a key conduit, sensing luminal and mucosal signals either directly or indirectly through enteroendocrine cells [221]. Experimental vagotomy has been shown to abolish or significantly attenuate microbiota-induced behavioral and neurochemical alterations, providing causal evidence that vagal pathways mediate microbiota–brain communication [222]. In addition, microbiota-derived products such as lipopolysaccharide (LPS) and peptidoglycan activate systemic immune responses, leading to the release of cytokines (e.g., IL-6, TNF-α) that can cross or signal across the BBB, thereby modulating CNS function and stress-related neural circuits [164]. Collectively, these pathways establish a multi-channel communication system through which the gut microbiota exerts regulatory control over brain function.

Stress-induced dysbiosis is not only compositional but also functionally pathogenic. It disrupts epithelial barrier integrity by downregulating Muc2 expression, reducing goblet cell numbers, and altering tight junction protein composition, thereby facilitating bacterial translocation and persistent immune activation [215,223]. This barrier breakdown increases exposure of mucosal immune cells to microbial-associated molecular patterns (MAMPs), activating pattern recognition receptors such as TLR4 and NOD-like receptors and triggering downstream pro-inflammatory signaling cascades [224].

Fecal microbiota transplantation (FMT) studies provide compelling causal evidence for the microbiota–gut–brain axis. Transfer of microbiota from IBD patients with comorbid depression into germ-free or specific pathogen–free mice induces both exacerbated colitis and depression-like behaviors, whereas microbiota from non-depressed IBD patients fails to reproduce these phenotypes [225]. Similarly, transplantation of microbiota derived from chronically stressed mice into healthy recipients results in behavioral disturbances and increased neuroinflammatory markers [226]. Notably, when subdiaphragmatic vagotomy is performed prior to microbiota transplantation, these effects are significantly attenuated, further supporting a critical role for parasympathetic (vagal) signaling in GBA communication [222].

Collectively, these findings demonstrate that the gut microbiota can simultaneously regulate intestinal inflammation and CNS-associated behaviors, supporting a shared mechanistic basis. The microbiota–gut–brain axis in IBD can therefore be conceptualized as a self-reinforcing loop, in which psychological stress induces dysbiosis, dysbiosis promotes barrier dysfunction and immune activation, and inflammation-derived signals feedback to the CNS to further modulate stress responsiveness. This closed-loop system provides a mechanistic explanation for the persistence and recurrence of IBD under conditions of chronic psychological stress.

## 3. Therapeutic Strategies in IBD: From Immune Suppression to Microbiota Modulation

### 3.1. Overview of the Therapeutic Landscape

**Figure 3:**
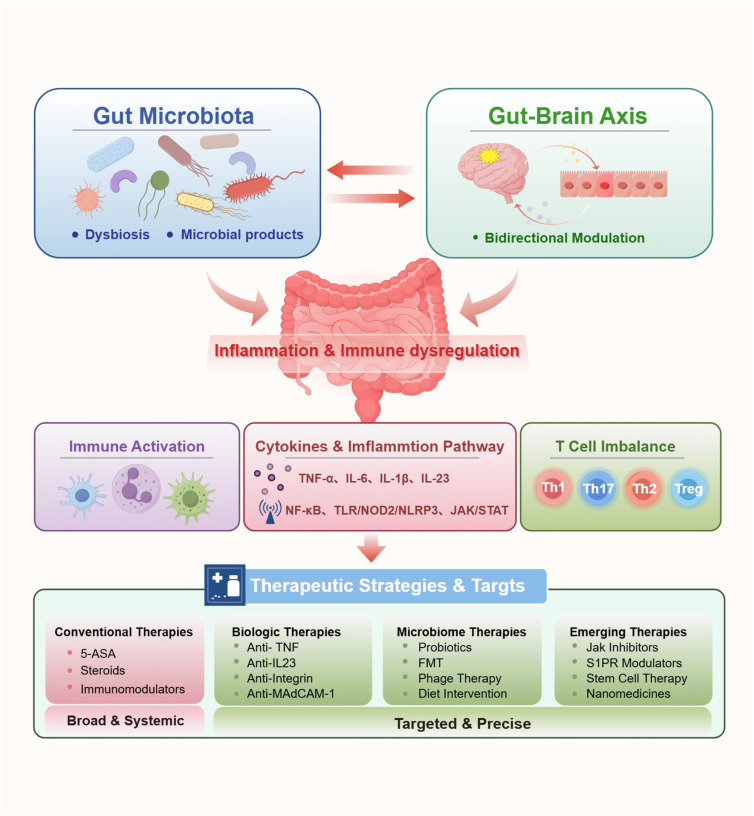
Core Pathogenic Mechanisms and Therapeutic Strategies in Inflammatory Bowel Disease. This figure illustrates the core mechanisms underlying IBD driven by microbial dysbiosis and gut-brain axis interactions. Intestinal dysbiosis and bidirectional regulation of the gut–brain axis act in concert to promote intestinal inflammation and immune dysregulation. This process involves activation of immune cells, release of pro-inflammatory cytokines, and engagement of key signaling pathways, along with T cell imbalance. Therapeutic strategies encompass conventional agents, biologics, microbiota-based therapies, and emerging approaches, reflecting a paradigm shift from broad-spectrum systemic treatment toward precision-targeted interventions. 5-ASA: 5-aminosalicylic acid; FMT: fecal microbiota transplantation; MAdCAM-1: mucosal addressin cell adhesion molecule-1; Jak: janus kinase; S1PR: sphingosine-1-phosphate receptor.

The therapeutic landscape of IBD has evolved from non-specific anti-inflammatory and immunosuppressive approaches to increasingly targeted and mechanism-based interventions [227] (see Figure 3). This shift reflects a growing recognition that effective treatment strategies should be aligned with key pathogenic processes, including immune dysregulation, epithelial barrier dysfunction, and gut microbiota alterations.

Immune dysregulation remains a central driver of IBD and is primarily targeted by conventional immunosuppressants, biologics, and small-molecule inhibitors. These therapies act by modulating key inflammatory pathways, such as TNF, IL-23/Th17, and JAK/STAT signaling [227]. Although biologics and immunosuppressants have significantly improved clinical remission rates, their mechanisms largely rely on systemic immune suppression, which is associated with an increased risk of infections and malignancies [228]. In addition, a substantial proportion of patients exhibit primary non-response or secondary loss of response, underscoring the need for alternative therapeutic paradigms [21]. In parallel, gut microbiota dysbiosis has emerged as a key factor influencing both immune responses and metabolic homeostasis [227]. Microbiota-targeted therapies aim to restore intestinal homeostasis rather than suppress immune activity. These approaches offer several theoretical advantages, such as preserving systemic immune competence, enabling multi-target regulation of metabolic and immune pathways, and potentially inducing durable disease modification [229]. However, their clinical application is currently limited by inter-individual variability, lack of standardization, and incomplete mechanistic understanding [229].

Collectively, this framework highlights a shift toward mechanism-oriented therapeutic strategies in IBD, in which treatments are increasingly tailored to specific pathogenic drivers. Based on this integrative perspective, the following sections discuss established immune-targeted therapies and emerging microbiota-based interventions, with an emphasis on their mechanistic basis, clinical efficacy, and therapeutic positioning.

### 3.2. Immune-Targeted and Small-Molecule Therapies

#### 3.2.1. JAK Inhibitors

Janus kinase inhibitors (JAKis) are a class of orally administered small-molecule agents that interfere with intracellular cytokine signaling through the JAK–STAT pathway, which mediates signal transduction for over 50 cytokines, including interleukins, interferons, and growth factors [230]. By inhibiting JAK phosphorylation and downstream STAT activation, these agents broadly modulate inflammatory pathways critical for intestinal homeostasis.

Currently approved JAK inhibitors for IBD include Tofacitinib, Filgotinib, and Upadacitinib [231]. Tofacitinib, a first-generation pan-JAK inhibitor with selectivity for JAK1 and JAK3 [232], received approval for moderate-to-severe UC in 2018 [233]. Phase III OCTAVE trials demonstrated superior efficacy compared with placebo for both induction and maintenance phases, with follow-up studies showing sustained benefits for up to 7 years [234]. However, Tofacitinib failed to meet primary endpoints in CD trials and remains unapproved for CD treatment [228]. In 2022, the UK approved the selective JAK1 inhibitor Filgotinib for the treatment of UC in patients with inadequate response or intolerance to conventional or biologic therapies [235]. Although phase II results in CD (FITZROY trial) were encouraging, the phase III DIVERSITY trial did not achieve its primary endpoints, limiting its indication to UC [236,237]. Upadacitinib, a second-generation selective JAK1 inhibitor, is currently the only JAK inhibitor approved for both UC and CD. Phase III trials (U-EXCEL, U-EXCEED, and U-ENDURE) demonstrated unparalleled efficacy in induction and maintenance treatment regardless of disease severity, with significantly higher clinical and endoscopic remission rates compared with placebo [231,238]. Network meta-analyses consistently rank upadacitinib as the most effective therapy for UC induction and CD maintenance [239,240,241]. Furthermore, it exhibits a rapid onset of action, with symptom improvement observed as early as 24 h in UC and within 14 days in CD, and demonstrates efficacy in extraintestinal manifestations (EIMs) and coexisting immune-mediated inflammatory diseases (IMIDs) [238,242].

#### 3.2.2. S1P Receptor Modulators

Sphingosine-1-phosphate receptor (S1PR) modulators represent another class of orally administered small molecule therapies that regulate lymphocyte trafficking by preventing lymphocyte egress from lymphoid tissues, thereby reducing intestinal inflammation. Ozanimod, the first selective S1PR1 and S1PR5 modulator approved for moderate-to-severe UC, demonstrated significantly higher efficacy compared with placebo during both induction (18.4% vs. 6.0%, *p* < 0.001) and maintenance (37.0% vs. 18.5%, *p* < 0.001) phases in clinical trials [243]. Another S1PR1 modulator, Etrasimod, also showed robust efficacy in the ELEVATE UC trials, with significantly higher response and remission rates than placebo [244]. In contrast, Amiselimod failed to demonstrate efficacy in phase IIa trials [245]. These agents hold particular value in patients with inadequate response to JAK inhibitors or biologics [246,247].

#### 3.2.3. Anti-Integrin and Anti-MAdCAM-1 Therapies

Lymphocyte trafficking to the intestinal mucosa is a critical step in IBD pathogenesis. The interaction between α4β7 integrin on T cells and mucosal addressin cell adhesion molecule-1 (MAdCAM-1) on intestinal endothelial cells mediates gut-specific homing [248]. Blocking this interaction selectively inhibits intestinal inflammation without systemic immunosuppression. The α4β7 integrin antibody Vedolizumab has been approved for both UC and CD. Subcutaneous Vedolizumab has demonstrated superior efficacy compared with placebo, achieving higher remission rates at 52 weeks (46% vs. 14%), while maintaining comparable effectiveness to the intravenous formulation [249,250]. Ontamalimab, a monoclonal antibody targeting mucosal addressin cell adhesion molecule-1 (MAdCAM-1), has also shown favorable efficacy in both UC and CD, providing an alternative therapeutic option for patients who are intolerant to integrin inhibitors [251,252].

#### 3.2.4. Anti-TNF and IL-23 Pathway Inhibitors

Anti-TNF agents remain a cornerstone of IBD therapy, with robust evidence supporting their efficacy. A Danish multicenter cohort study reported that, approximately 75% of UC patients and 80% of CD patients achieved a clinical response during long-term follow-up [253]. Advances such as subcutaneous infliximab (CT-P13 SC) and oral TNF inhibitors (e.g., AVX-470, OPRX-106) represent innovations in anti-TNF therapy, substantially improving convenience and safety in IBD treatment [254,255,256,257]. Furthermore, nanotechnology-based anti-TNF antibody delivery systems further enhance targeted drug accumulation in inflamed tissues [258].

Second-generation IL-23p19 inhibitors represent a major advancement in targeted therapy. Mirikizumab has demonstrated sustained efficacy for up to 152 weeks in UC patients who are refractory to anti-TNF or JAK inhibitors [259]. Risankizumab has been approved for CD, with an induction regimen of 600 mg administered intravenously at weeks 0, 4, and 8, followed by maintenance therapy with 360 mg subcutaneously every 8 weeks [260]. Guselkumab and Brazikumab have also shown promising results in clinical trials [261,262]. These agents offer more selective immunomodulation by specifically targeting the IL-23/Th17 axis.

#### 3.2.5. Cell-Based Therapies

Mesenchymal stem cells (MSCs) exert immunomodulatory and tissue-repair functions and have demonstrated significant efficacy in IBD, increasing clinical remission rates by 29.9% and fistula healing rates by 35.8% [263]. Adipose- and bone marrow-derived MSCs are the most commonly used sources [264].

Hematopoietic stem cell transplantation (HSCT) offers a potential curative approach for refractory CD by resetting the immune system but is associated with substantial risks, including infection and hematologic toxicity [265]. Notably, allogeneic HSCT has shown remarkable efficacy in monogenic IBD, achieving drug-free remission in 92% of patients [266].

### 3.3. Microbiota-Targeted Therapies

The gut microbiota plays a central role in IBD pathogenesis, with dysbiosis characterized by reduced diversity, depletion of beneficial commensals, and expansion of pro-inflammatory pathobionts [68,69]. Importantly, these alterations are not merely compositional but also functional, involving disrupted production of key microbial metabolites and impaired host–microbiome signaling.

#### 3.3.1. Fecal Microbiota Transplantation (FMT)

FMT represents a prototypical microbiota-restoration strategy, involving transfer of a functionally intact microbial community—including bacteria, archaea, fungi, and viruses—from healthy donors to recipients, thereby restoring microbial diversity, functional redundancy, and host–microbiome metabolic interactions [267]. FMT has demonstrated remarkable efficacy in recurrent *Clostridioides difficile* infection, with remission rates exceeding 85% and confirmed long-term safety over ≥2 years of follow-up [268]. In UC, randomized controlled trials have reported clinical remission rates of 30–40% and endoscopic improvement in 25–35% of patients, with meta-analyses confirming significant benefits over placebo at 8–12 weeks [269,270,271]. Notably, single-dose fresh FMT has been shown to induce sustained drug-free remission and promote histological healing via suppression of TNF-α/IL-23 signaling and restoration of epithelial barrier integrity [272,273].

Washed microbiota transplantation (WMT), which removes >99% of non-microbial components while preserving viable microbes and extracellular vesicles, has demonstrated improved efficacy in CD and reduced long-term healthcare costs compared with infliximab-based strategies [274,275].

Emerging evidence highlights the role of the gut virome in modulating therapeutic outcomes. Fecal virome transplantation (FVT) selectively enriches Caudovirales bacteriophages and reshapes bacterial networks through targeted depletion of pathogens and expansion of commensals [276,277]. However, key questions remain regarding viral stability, host adaptation, and long-term safety, necessitating further longitudinal studies.

#### 3.3.2. Phage Therapy

Phage therapy represents a precision “subtractive” strategy that selectively eliminates pathogenic bacteria while preserving commensal diversity. Compared with broad-spectrum antibiotics, which can exacerbate dysbiosis and inflammation, bacteriophages offer strain-level specificity and reduced ecological disruption [278].

In murine colitis models, oral administration of rationally designed phage cocktails reduced Klebsiella load without altering α-diversity, accompanied by significant attenuation of colonic inflammation. Importantly, no resistant mutants emerged during 28 days of treatment due to synergistic phage combinations [279]. Engineered phage platforms have further expanded targeting capabilities, enabling recognition of multiple bacterial receptors [280]. Nevertheless, clinical translation faces challenges, including phage resistance, gastric inactivation, and delivery optimization. Strategies such as encapsulation and rotating phage libraries are being developed to overcome these limitations [281,282,283].

#### 3.3.3. Engineered Probiotics

Engineered probiotics leverage synthetic biology to transform commensal bacteria into programmable therapeutic platforms capable of localized drug delivery and microenvironment-responsive activity [284]. Examples include EcN strains engineered to express antioxidant enzymes (KatG, SodA), which reduce oxidative stress and promote colonization of beneficial anaerobes, thereby alleviating inflammation [285]. Advanced systems such as POSR@EcN enable inflammation-triggered release of gasotransmitters (CO and H_2_S), enhancing barrier function and modulating G-related outcomes [286]. Other platforms, including EcN-TRP@A/G and EcN-SP composites, further demonstrate the potential of engineered microbes in regulating metabolic pathways and improving colonization efficiency [287,288].

#### 3.3.4. Anti-Adhesion and Anti-Invasion Therapies

Targeting pathogen–host interaction represents a novel therapeutic paradigm. AIEC, implicated in CD, adheres to epithelial cells via FimH–CEACAM6 interactions, triggering NF-κB activation and increasing postoperative recurrence risk [50,53,289,290]. TAK-018, a selective FimH antagonist, blocks bacterial adhesion and has demonstrated favorable safety and pharmacodynamic effects in phase Ib trials, including reduced AIEC load and delayed endoscopic recurrence [291]. Biomaterial-based approaches, such as AIEC-targeting microgels, enable physical sequestration of pathogens without systemic immune activation, representing a drug-free therapeutic strategy [292].

#### 3.3.5. Dietary Interventions

Dietary interventions provide a physiologically grounded approach to modulating the gut microbiota. Diets such as Crohn’s Disease Exclusion Diet (CDED), Mediterranean diet, low-FODMAP, Specific Carbohydrate Diet (SCD), and Whole-Food, Plant-Based Diet (WFPBD), exert selective pressure on microbial communities by altering substrate availability [293]. Western diets promote dysbiosis, while low-FODMAP diets improve symptoms but may reduce beneficial bacteria (such as *Bifidobacterium adolescentis* and *B. longum*), underscoring the need for personalized nutrition [294]. Exclusive enteral nutrition (EEN) induces mucosal healing and modulates inflammation via NF-κB inhibition and microbiota alterations [295,296,297]. Time-restricted feeding (TRF) further integrates circadian regulation with microbial metabolism, significantly reducing systemic levels of IGF-1, IL-6, TNF-α, and IL-1, while enriching SCFA-producing taxa including *Rikenellaceae Lactobacillus*, and *Ruminococcus* [298,299].

Beyond these dietary patterns, plant-derived natural products have emerged as key modulators of the gut microbiota–host metabolic axis. Plant polysaccharides act as prebiotic-like components, generating bioactive metabolites (SCFAs, secondary bile acids, tryptophan derivatives) that regulate host immunity and inflammation [300]. Similarly, the alkaloid berberine promotes beneficial bacteria (Bacteroides, Bifidobacterium, Lactobacillus) while suppressing pathogenic taxa, and exerts anti-inflammatory effects via NF-κB suppression and barrier protection [301]. Given their low oral bioavailability, both polysaccharides and berberine exert therapeutic effects primarily through direct interaction with the gut microbiota, positioning them as paradigmatic microbiota-dependent interventions [300]. These approaches provide new approaches for the combined regulation of metabolism and microecology in IBD.

## 4. Conclusions

In summary, IBD arises from a highly integrated network of host–microbial–immune interactions, in which gut microbiota dysbiosis functions as a central pathogenic hub. The convergence of reduced microbial diversity, loss of immunoregulatory commensals, and expansion of pro-inflammatory pathobionts disrupts epithelial barrier integrity, facilitates microbial translocation, and perpetuates chronic mucosal inflammation. Importantly, these local intestinal processes are further amplified by bidirectional signaling along the GBA, which links neuroendocrine stress responses to immune dysregulation and microbial imbalance, thereby contributing not only to disease progression but also to systemic and behavioral comorbidities.

Advances in multi-omics technologies, biomarker discovery, and AI-assisted diagnostics are refining disease stratification and enabling earlier, more precise assessment of disease activity and therapeutic response. Within this evolving landscape, therapeutic strategies are undergoing a conceptual transition from non-specific immunosuppression toward mechanism-based interventions. Microbiota-targeted approaches—including standardized fecal microbiota transplantation, rationally designed live biotherapeutic products, precision bacteriophage therapy, and microbiome-informed dietary modulation—offer promising avenues to restore microbial homeostasis and recalibrate host–microbe interactions.

Despite these advances, several challenges remain, including inter-individual heterogeneity in microbiome composition, variable therapeutic responses, and the need for standardized protocols and long-term safety data. Future research should prioritize integrative, longitudinal studies that combine microbiome profiling, host genetics, immune phenotyping, and neuroendocrine parameters to delineate causal pathways and identify robust predictive biomarkers. Ultimately, a deeper mechanistic understanding of the microbiota–gut–brain axis will be essential for translating these insights into clinically actionable strategies. Such efforts will pave the way toward truly personalized medicine in IBD, where targeted modulation of the intestinal ecosystem and its systemic connections can achieve sustained remission and improved patient outcomes.

## Data Availability

All raw data and code are available upon request.
